# Comparison of safety and short-term outcomes of septostomy and direct tunnel passage techniques in percutaneous patent foramen ovale closure: a multicenter experience

**DOI:** 10.3389/fcvm.2026.1741158

**Published:** 2026-03-26

**Authors:** Senol Coskun, Selma Kenar Tiryakioğlu, Didar Mirzamidinov, Duygu Inan, Alev Kilicgedik, Tayfun Sahin, Teoman Kilic

**Affiliations:** 1 Department of Cardiology, Kocaeli University of Health and Technology, Kocaeli, Turkiye; 2 Department of Cardiology, Bursa City Hospital, Bursa, Turkiye; 3 Structural Heart Interventions Unit, Department of Cardiology, Kocaeli University School of Medicine, Kocaeli, Turkiye; 4 Department of Cardiology, Cam and Sakura City Hospital, Istanbul, Turkiye

**Keywords:** atrial septostomy, complications, cryptogenic stroke, patent foramen ovale, PFO closure, procedural safety

## Abstract

**Background:**

Percutaneous closure of a patent foramen ovale (PFO) is an established therapeutic strategy for patients with cryptogenic stroke (CS). This study aimed to compare the procedural safety, efficacy, and short-term clinical outcomes of atrial septostomy vs. standard direct tunnel passage techniques for PFO closure.

**Methods:**

We retrospectively analyzed consecutive patients who underwent PFO closure for CS at four centers between January 2010 and December 2020. Demographic characteristics, vascular risk factors, Risk of Paradoxical Embolism (RoPE) scores, PFO anatomical features, procedural details, and clinical outcomes were compared between patients treated with atrial septostomy and those undergoing direct tunnel passage. Continuous variables are presented as mean ± standard deviation or median [interquartile range], and categorical variables as counts and percentages.

**Results:**

A total of 246 patients were included (mean age 40.5 ± 9.8 years), of whom 69.9% presented with ischemic stroke and 30.1% with transient ischemic attack (TIA). Atrial septostomy was performed in 106 patients (43.1%), while 140 patients (56.9%) underwent direct tunnel passage. Baseline clinical characteristics were comparable between groups; however, the septostomy group exhibited significantly more complex PFO anatomy, including atrial septal aneurysm (69.8% vs. 39.3%), septal hypermobility (62.3% vs. 34.3%), and spontaneous shunting at rest (85.8% vs. 50.7%; all *p* < 0.001). Procedure duration was similar between groups (41.1 ± 10.2 vs. 40.7 ± 13.8 min; *p* = 0.83), and technical success was achieved in nearly all cases. No major procedural complications occurred. Minor adverse events were infrequent and comparable (8.4% vs. 4.3%; *p* = 0.20). At six-month follow-up, residual shunt rates were numerically lower in the septostomy group (6.6% vs. 11.4%; *p* = 0.27). No TIAs were observed in the septostomy group, whereas a 3.6% incidence was noted in the direct passage group (*p* = 0.08). New-onset atrial arrhythmia occurred in two patients (1.9%) in the septostomy group and in none of the direct passage group (*p* = 0.18).

**Conclusion:**

Despite being applied in patients with more complex PFO anatomy, atrial septostomy demonstrated comparable procedural safety, technical success, and short-term efficacy to standard direct tunnel passage. Tunnel length emerged as an independent predictor of residual shunt, regardless of closure technique. In experienced centers, atrial septostomy appears to be a safe and practical alternative for anatomically challenging PFO closures.

## 1 Introduction

PFO is a common congenital cardiac structure, present in approximately 25% of the general population as a remnant of fetal circulation. Although typically benign, PFO can serve as a conduit for paradoxical embolism and play an etiologic role in a subset of cryptogenic stroke (CS) cases, particularly among young adults (1). The pathophysiology of paradoxical embolism involves the passage of a thrombus formed in the venous system through the PFO tunnel during transient elevations in right atrial pressure—such as during a Valsalva maneuver—allowing it to enter the systemic circulation and occlude cerebral arteries (2). PFO is detected in nearly 50% of patients under 60 years of age with cryptogenic stroke, approximately twice the prevalence observed in the general population (3).

Several risk stratification tools have been developed to better define the causal role of PFO in stroke. The most widely used, the Risk of Paradoxical Embolism (RoPE) score, estimates the likelihood that a detected PFO is causally related to stroke, based on clinical variables such as age and conventional vascular risk factors. A higher RoPE score indicates a greater probability of a pathogenic association, particularly in younger patients (4).

Percutaneous PFO closure is an established therapeutic option for patients with PFO-related CS. Multiple randomized controlled trials in recent years have demonstrated that percutaneous closure, in well-selected patients, is superior to medical therapy alone for secondary stroke prevention (5–7). Based on this evidence, international guidelines recommend percutaneous PFO closure in patients aged 18–60 years with cryptogenic stroke and suitable anatomical features (8).

Transcatheter closure can be performed with high success rates in experienced centers. Currently, two main procedural approaches are employed: the standard technique, involving direct passage through the PFO tunnel for device delivery, and the transseptal technique, in which a controlled atrial septostomy is created adjacent to the PFO tunnel. The standard method entails advancing a guidewire from the right atrium through the PFO into the left atrium, followed by delivery sheath and occluder placement. However, long-tunnel and complex PFO anatomies may pose significant procedural challenges. In the presence of a prominent Eustachian ridge, an excessively redundant Chiari network, a thick and highly mobile septum primum, or an elongated long PFO tunnel, direct tunnel crossing with conventional wire–catheter manipulation may be time-consuming, unsuccessful, or potentially traumatic. Long or tortuous PFO tunnels, coexisting atrial septal aneurysm (ASA), small PFO openings, or prominent Eustachian valve/Chiari network structures can hinder wire passage and device positioning (9–11).

In anatomically complex cases, creating a controlled atrial septostomy adjacent to the PFO tunnel can facilitate device delivery without increasing the risk of septal tearing or device instability. This technique involves forming an artificial fenestration in the septum primum, through which the device is advanced into the left atrium (10–13). Septostomy may also serve as a “bail-out” technique following unsuccessful direct crossing attempts. However, as the technique intentionally creates a new interatrial defect, it may be associated with additional risks, including cardiac tamponade, persistent residual shunt, atrial arrhythmias, or device malposition. Therefore, establishing its real-world safety and efficacy remains essential (10–13).

To address this knowledge gap, the present study sought to compare the procedural safety, short-term efficacy—assessed by residual shunt rates—and clinical outcomes, including recurrent neurological events, between atrial septostomy and standard direct crossing techniques in a homogeneous cohort of patients undergoing PFO closure for secondary prevention after cryptogenic stroke.

## 2 Methods

### 2.1 Study population

This multicenter, retrospective, observational cohort study included patients who underwent interatrial shunt closure in four high-volume cardiology centers between January 2010 and December 2020. Data from 249 consecutive patients were reviewed. Patients who underwent percutaneous PFO closure for ischemic stroke, transient ischemic attack (TIA), or other secondary prevention indications as determined by the operator were included. In cases where a patient underwent more than one procedure (e.g., device removal and re-implantation), each intervention was analyzed separately. Patients with incomplete clinical data were excluded. Three patients treated for primary atrial septal defect (ASD) were also excluded, resulting in a final study cohort of 246 patients. The study protocol was approved by the Kocaeli University Ethics Committee (Approval Date: 25.09.2025, Reference No: GOKAEK-2025/19/09, Project ID: 2025/484) and conducted in accordance with the Declaration of Helsinki.

### 2.2 Group definitions and data collection

Patients were categorized according to the PFO closure technique used:

- Septostomy Group (Group 1): Patients who underwent atrial septostomy due to technical difficulty in crossing the PFO tunnel before device deployment.
- Direct Passage Group (Group 2): Patients in whom the guidewire and device were advanced directly through the PFO tunnel.

Demographic and clinical variables, stroke-related characteristics, anatomic findings, procedural details, and follow-up outcomes were collected. Demographic data included age, sex, height, weight, and body mass index (BMI). Cardiovascular risk factors such as hypertension, diabetes mellitus, smoking, hyperlipidemia, and coronary artery disease were recorded. Neurological history included the index event (ischemic stroke or TIA), number of recurrent strokes, and the presence of lacunar infarction. The Risk of Paradoxical Embolism (RoPE) score was also assessed.

Echocardiographic and angiographic data included interatrial septal hypermobility (aneurysmal motion), PFO tunnel length, presence of ASA, spontaneous right-to-left shunt at rest, hybrid defects (PFO with small ASD), septal thickness, and the presence of a Eustachian ridge or Eustachian valve/Chiari network. Shunt severity was graded by the number of bubbles appearing in the left atrium during contrast (agitated saline) study as mild (3–10 bubbles), moderate (10–30), or severe (>30). These anatomical variables were coded as binary (present/absent).

Procedural data included device type (e.g., Amplatzer PFO Occluder, Occlutech Figulla flex II and Lifetech Ceraflex), device size (mm), total procedure duration (minutes), and access technique. Procedural or periprocedural complications were classified as none (0), minor (1; clinically insignificant or managed conservatively), or major (2; life-threatening or requiring urgent intervention).

### 2.3 Endpoints

The primary safety endpoint was the composite rate of major periprocedural complications within 30 days, including death, cardiac tamponade or perforation, device embolization, major vascular complication requiring surgery, or procedure-related stroke.

The primary efficacy endpoint was the presence of a significant residual shunt at six months, defined as >10 microbubbles entering the left atrium during contrast transthoracic echocardiography (TTE) with Valsalva maneuver. Secondary endpoints included recurrent ischemic stroke, recurrent TIA, new-onset atrial fibrillation, and minor procedural complications (e.g., groin hematoma).

### 2.4 Statistical analysis

Descriptive statistics were presented as mean ± standard deviation (SD) or median [interquartile range, IQR] for continuous variables, and as frequencies and percentages for categorical variables. Continuous variables were compared using the Student's t-test for normally distributed data or the Mann–Whitney U test for non-normally distributed data. Categorical variables were compared using the Chi-square test or Fisher's exact test, as appropriate. Two-tailed *p* values <0.05 were considered statistically significant. All analyses were performed using the R Statistical tool environment.

## 3 Results

### 3.1 Patient population

During the 10-year study period, a total of 246 patients were included. The septostomy technique was used in 106 patients (43.1%) and direct crossing method was used in 140 patients (56.9%). Indication for PFO closure was ischemic stroke in 172 patients and transient ischemic attack (TIA) in 74 patients. Baseline demographic and clinical characteristics between the septostomy group and direct crossing groups are summarized in Table 1. The mean ages were similar between the septostomy and direct passage groups (40.3 ± 10.7 vs. 40.7 ± 9.2 years, *p* = 0.79). The proportion of female patients was comparable (51.9% vs. 47.9%, *p* = 0.53). BMI values did not differ significantly (25.9 ± 3.4 vs. 26.4 ± 4.0 kg/m^2^, *p* = 0.35). Likewise, the prevalence of hypertension, diabetes, smoking, hyperlipidemia, and coronary artery disease showed no significant difference (*p* > 0.05) (Table 1). The mean value of RoPE score was also similar between the two groups (6.8 ± 1.7 vs. 7.0 ± 2.0, *p* = 0.46)

**Table 1:** Basic demographic and clinical characteristics of the groups.

Characteristic	Septostomy Group (*n* = 106)	Direct Passage Group (*n* = 140)	*p*-value
Age (years), mean ± SD	40.4 ± 10.6	40.6 ± 9.2	0.83
Female sex, *n* (%)	55 (51.9%)	67 (47.9%)	0.53
BMI (kg/m^2^), mean ± SD	25.9 ± 3.4	26.4 ± 4.0	0.35
Hypertension, *n* (%)	29 (27.4%)	36 (25.7%)	0.81
Diabetes, *n* (%)	12 (11.3%)	21 (15.0%)	0.38
Smoking, *n* (%)	36 (34.0%)	57 (40.7%)	0.28
Hyperlipidemia, *n* (%)	21 (19.8%)	36 (25.7%)	0.27
CAD, *n* (%)	10 (9.4%)	15 (10.7%)	0.76
Stroke indication, *n* (%)	87 (82.1%)	85 (60.7%)	<0.001
TIA indication, *n* (%)	19 (17.9%)	55 (39.3%)	<0.001
RoPE Score, mean ± SD	7.06 ± 2.01	6.88 ± 1.71	0.46

BMI, Body mass index; CAD, Coronary artery disease; TIA, Transient ischemic attack; RoPE, Risk of paradoxical embolism; SD, Standard deviation; IQR, Interquartile range.

### 3.2 Anatomical characteristics

Comparison of anatomical features of patent froman ovale between the septostomy group and direct passage group is summaried in Table 2. Anatomical features influencing procedural strategy revealed that patients in the septostomy group exhibited more complex atrial septal anatomy. Specifically, atrial septal aneurysm (ASA) was significantly more frequent in the septostomy group (69.8% vs. 39.3%, *p* < 0.001), as was septal hypermobility (62.3% vs. 34.3%, *p* < 0.001). PFO tunnel length did not differ significantly between groups [10 mm (IQR 8–12) vs. 10 mm 8–13, *p* = 0.06]. However, spontaneous right-to-left shunt at rest was considerably more common in the septostomy group (85.8% vs. 50.7%, *p* < 0.001), and a thick septal structure was also more prevalent (33.0% vs. 19.3%, *p* = 0.017). No significant differences were found regarding the presence of a prominent Eustachian ridge (29.9% vs. 25.2%, *p* = 0.47) or Eustachian valve/Chiari network (17.8% vs. 22.3%, *p* = 0.43).

**Table 2:** Comparison of anatomical features of groups.

Anatomic Feature	Septostomy Group (*n* = 106)	Direct Passage Group (*n* = 140)	*p*-value
Septal hypermobility, *n* (%)	66 (62.3%)	48 (34.3%)	<0.001
Tunnel length (mm), med [IQR]	10 [8–12]	10 [8–13]	0.06
Atrial septal aneurysm, *n* (%)	74 (69.8%)	55 (39.3%)	<0.001
Shunt at rest, *n* (%)	91 (85.8%)	71 (50.7%)	<0.001
Hybrid defect, *n* (%)	16 (15.1%)	8 (5.7%)	0.018
Thick septum, *n* (%)	35 (33.0%)	27 (19.3%)	0.017
Prominent Eustachian ridge, *n* (%)	31 (29.2%)	35 (25.0%)	0.47
Eustachian valve/Chiari network, *n* (%)	19 (17.9%)	31 (22.1%)	0.43

Med, median; IQR, interquartile range.

### 3.3 Procedural details

Procedural characteristics and six-month follow-up outcomes are summarized in Table 3. Various PFO closure devices were used in both cohorts. The Amplatzer PFO occluder was the most commonly implanted device, utilized in 67.3% of patients in the septostomy group and 53.2% in the direct passage group (*p* = 0.04). Occlutech Figulla flex II devices were used in 32.7% and 40.3% of patients, respectively (*p* = 0.28), while the Lifetech Ceraflex device was exclusively used in the direct passage group (6.5% vs. 0%, *p* = 0.006, Table 3). The mean device size was significantly larger in the septostomy group compared with the direct passage group (30.9 ± 4.9 mm vs. 25.4 ± 4.2 mm, *p* < 0.001).

**Table 3:** Procedural details and 6-month short-term outcomes.

Variable	Septostomy Group (*n* = 106)	Direct Passage Group (*n* = 140)	*p*-value
Procedural Data			
Device model (Amplatzer), *n* (%)	72 (67.9%)	74 (52.9%)	0.04
Device model (Occlutech Figulla flex II), *n* (%)	34 (32.1%)	57 (40.7%)	0.28
Device model (Lifetech Ceraflex), *n* (%)	0 (0%)	9 (6.4%)	0.006
Device size (mm), mean ± SD	30.9 ± 4.9	25.4 ± 4.2	<0.001
Procedure duration (min), mean ± SD	41.1 ± 10.2	40.7 ± 13.8	0.83
Procedural and Clinical Outcomes			
Any complication, *n* (%)	10 (9.4%)	6 (4.3%)	0.15
Major complication, *n* (%)	0 (0%)	0 (0%)	1.00
Minor complication, *n* (%)	9 (8.4%)	6 (4.3%)	0.20
Residual shunt (6th month), *n* (%)	7 (6.6%)	16 (11.4%)	0.27
Post-procedural stroke, *n* (%)	0 (0%)	0 (0%)	1.00
Post-procedural TIA, *n* (%)	0 (0%)	5 (3.6%)	0.08
Arrhythmia (AF/AFl), *n* (%)	2 (1.9%)	0 (0%)	0.18

SD, Standard deviation; min, minutes; TIA, Transient ischemic attack; AF, Atrial fibrillation; AFl, Atrial flutter.

Mean procedure duration was comparable between the two groups (41.2 ± 10.3 vs. 40.9 ± 13.9 min, *p* = 0.85). No major periprocedural complications were observed in either group (0% vs. 0%). Minor complications—primarily access-site hematomas and transient arrhythmic episodes—were observed in 8.4% and 4.3% of patients, respectively (*p* = 0.20), all of which resolved completely with conservative management. The overall rate of procedural complications was 9.3% (*n* = 10) in the septostomy group and 4.3% (*n* = 6) in the direct passage group (*p* = 0.15).

### 3.4 Follow-up outcomes of two groups

At six-month follow-up, the rate of significant residual shunt—the primary efficacy endpoint—was similar between the septostomy and direct passage groups (6.6% vs. 11.4%, *p* = 0.27). No recurrent ischemic strokes were observed in either group; however, five transients ischemic attack (TIA) events (3.6%) occurred in the direct passage group. A detailed re-evaluation of patients treated with the direct tunnel crossing method revealed that both stroke and TIA were significantly more frequent as baseline indications for PFO closure in this group. In this context, although the difference did not reach statistical significance, the observed numerical variation in recurrent neurological events—consistent with previously published studies—may be related to differences in baseline clinical indications, particularly the higher prevalence of stroke and TIA in the direct passage group.

New-onset atrial arrhythmias, including atrial fibrillation or flutter, were detected in two patients (1.9%) in the septostomy group and in none of the patients in the direct passage group (*p* = 0.18). These episodes were transient and successfully managed with medical therapy, with no progression to persistent or chronic atrial fibrillation.

## 4 Discussion

In this retrospective multicenter study, the short-term outcomes of two different percutaneous closure strategies for PFO in patients with cryptogenic stroke were compared. Our findings indicate that despite being applied in anatomically more challenging cases, the septostomy-assisted technique achieved procedural safety outcomes comparable to those of the standard direct passage technique. In other words, the rates of major complications and post-procedural adverse neurological events did not differ significantly between the two approaches. These results suggest that, when performed by experienced operators, septostomy can be a safe and feasible alternative in selected patients.

Septostomy is typically reserved for complex anatomical cases or when standard wire passage through the PFO tunnel fails (14). In our cohort, the septostomy group exhibited a significantly higher prevalence of ASA, septal hypermobility, thick septal tissue, and spontaneous resting shunt—features known to complicate direct PFO access. Nevertheless, the absence of major complications in this group is noteworthy. Large registries have reported serious procedural complication rates below 1% for PFO closure (15, 16). For instance, in a real-world series of 1,887 patients with stroke/TIA, the in-hospital adverse event rate was 7.0% (16). The complication rates observed in our study (9.3% vs. 4.3%) are consistent with these findings, and the intergroup difference was not statistically significant. The slightly longer procedural duration observed in the septostomy group may be related with the additional step of creating an atrial septostomy. Despite the additional procedural step, mean procedure duration was comparable between the two groups (41.2 ± 10.3 vs. 40.9 ± 13.9 min, *p* = 0.85). This finding may be explained by the high level of operator expertise at participating centers, particularly in structural heart interventions requiring transseptal access, such as paravalvular leak closure, mitral balloon valvuloplasty, and transcatheter edge-to-edge repair. Prior studies have also noted that this maneuver may prolong the procedure by only a few minutes (12, 14). Although minor complications (e.g., bleeding, transient arrhythmia) were slightly more frequent in the septostomy group, this difference did not reach statistical significance. These all results suggest that, despite being preferentially used in patients with more complex PFO anatomy, the septostomy technique does not increase procedural risk and appears to be as safe as the direct passage approach.

At six-month follow-up, residual shunt rates were similar between the two groups (6.5% in the septostomy group vs. 11.5% in the direct passage group, *p* = 0.27), demonstrating that both techniques provide effective closure. Previous studies have shown that small residual shunts after PFO closure are not uncommon. Some studies have suggested that these residual shunts often decrease or disappear over time and are not associated with a significant clinical impact in most patients, whereas others have proposed that residual shunts may increase the risk of ischemic events (17–19).

Similar rate of residual shunts between the two groups in our cohort may also be evaluated as the success of the septostomy technique when it is properly performed. Despite a higher rate of complex anatomy and larger device sizes in the septostomy group, residual orifice rate at sixth month was not found to be different. However, given that residual shunts may also increase long-term stroke recurrence risk (17, 19), careful monitoring and secondary interventions—such as repeat closure or anticoagulation—should be considered when necessary.

When post-procedural neurological events are examined, the recurrent stroke rate is zero in both techniques, and the results are extremely positive. The rate of stroke or TIA development in the early period after closure is also very low in the literature, generally at levels of 1%-2% (16). In our series as well, no stroke events were observed in the 6-month follow-up. As a minor difference, post-procedural TIA occurred in 3.6% (5 patients) of the patients in the direct passage group. In general, secondary stroke/TIA rates in both groups are very low after the closure procedure, and this finding is consistent with previous studies supporting the efficacy of PFO closure (17). Furthermore, it is significant that serious arrhythmias such as atrial fibrillation emerged at an extremely low rate in both groups (only 1.9% in the septostomy group). As it is known that, one of the most common complications after PFO closure is new-onset atrial fibrillation, which is reported at rates of approximately 3%-8% in large series (20, 21). The fact that this rate remained very low in our study (0.8% in total) can be attributed to our patient population being relatively young.

### 4.1 Limitations

This study has several important limitations. First, its retrospective design relied on data extracted from existing records, which may have introduced information and selection biases related to incomplete or non-standardized documentation. In particular, the specific procedural indications for septostomy—such as failed wire passage or challenging anatomical features—were not consistently documented. Second, as the data were derived from four high-volume centers, the findings may reflect center-specific expertise and procedural preferences, potentially limiting the generalizability of the results. Third, although the sample size was moderate, the study may have been underpowered to detect differences in infrequent outcomes, including major complications or recurrent stroke. In addition, the classification of adverse events as “minor” or “major” was based on institutional criteria, which may not fully correspond to standardized definitions. Finally, the follow-up period was limited to six months, precluding assessment of long-term outcomes such as late stroke recurrence or device-related complications.

## 5 Conclusion

In this retrospective analysis of 246 patients undergoing percutaneous PFO closure for cryptogenic stroke, atrial septostomy—despite being preferentially used in anatomically complex cases—demonstrated short-term safety and efficacy comparable to the standard direct passage technique. Notably, although challenging anatomical features such as atrial septal aneurysm, septal hypermobility, and spontaneous shunting were more prevalent in the septostomy group, no increase in major complications or residual shunt rates was observed at six-month follow-up. These findings indicate that, when performed in experienced centers, septostomy-assisted PFO closure may serve as a safe and practical alternative for selected patients with complex interatrial septal anatomy. However, given the inherent limitations of the retrospective study design, prospective randomized multicenter trials are warranted to further clarify the long-term safety and efficacy of different access strategies.

## Data Availability

The original contributions presented in the study are included in the article/Supplementary Material, further inquiries can be directed to the corresponding author.
